# Drug-induced metabolic remodeling of immune cell repertoire generates an effective broad-range antimicrobial effect

**DOI:** 10.21203/rs.3.rs-7077811/v1

**Published:** 2025-07-29

**Authors:** Bhupesh Kumar Prusty, Claudia Hollmann, Eun Chan Park, Zheng Liu, Faye Nourollahi, Georgy Nikolayshvili, Jonathan Dietz, Emils Bašēns, Mehul Vora, Trushnal Waghmare, Tongbin Li, Fabian Imdahl, Christopher Rongo

**Affiliations:** Riga Stradins University; Julius-Maximilians University of Würzburg; The Waksman Institute, Rutgers The State University of New Jersey; Julius-Maximilians University of Würzburg; The Waksman Institute, Rutgers The State University of New Jersey; Riga Stradins University; The Waksman Institute, Rutgers The State University of New Jersey; Riga Stradins University; ModOmics Ltd; Riga Stradins University; AccuraSciences; Helmholtz Institute for RNA-based Infection Research; The Waksman Institute, Rutgers The State University of New Jersey

## Abstract

Multiple mechanisms of immunity must be coordinated to defend against a comprehensive range of pathogens; however, the mechanisms by which broad-spectrum antipathogens act remain largely elusive. Here, we employed systems biology approaches to understand the organization of human immune cells at the single-cell level, as well as their reorganization in response to K21, a silane derivative effective against viral, bacterial, and fungal infections. K21 induced pro-inflammatory pathways in M1 and M2c macrophages without altering cytokine secretion, decreased a specific subtype of M1 macrophages and CXCL4-induced M2-like macrophages, and improved mitochondrial health by enhancing mitochondrial recycling via mitophagy. Similar treatment of the *in vivo* model organism *C. elegans* induced mitophagy and extended lifespan, suggesting evolutionary conservation of mechanism. Our work demonstrates that a drug that remodels mitochondria and metabolism can shape the immune cell repertoire, which could aid the development of more effective antimicrobials and prevent the emergence of drug-resistant pathogens.

## INTRODUCTION

Macrophages are the key mediators of the innate immune system, which provides an early line of defense once a pathogen breaches barrier tissues to enter the body. Macrophages exhibit immense variability and plasticity in function and differentiation, enabling these cells to respond to a broad range of pathogens, including viruses, bacteria, and fungi, as well as adapt to diverse microenvironments and various stages of infection and healing^1^. Naïve macrophages (non-polarized M0) have a relatively quiescent metabolism that relies more on fatty acid beta-oxidation than glycolysis for energy production^2^. M0 macrophages can differentiate into M1 (classically activated) macrophages or M2 (alternatively activated macrophages) depending on exposure to specific TLR agonists and cytokines^3^, with the specific macrophage phenotype dictating whether the innate immune response is antimicrobial and pro-inflammatory (for M1), or more anti-inflammatory and thus favorable for tissue repair (for M2). Metabolic differences are important aspects of M1 versus M2 polarization, as M1 macrophage metabolism is characterized by elevated glycolysis, pentose phosphate pathway (PPP) flux, and fatty acid synthesis, whereas M2 macrophage metabolism is characterized by elevated fatty acid oxidation and oxidative phosphorylation (OXPHOS)^4–7^. The metabolic reprogramming of macrophages is a crucial aspect of their functional plasticity; however, we lack a comprehensive understanding of how these metabolic changes are mediated and how they can be regulated to provide therapeutic benefits.

Traditionally macrophage differentiation has been analyzed through a combination of cytokine-based stimulatory factor treatment (e.g., GM-CSF and M-CSF to induce M1 and M2 naïve macrophages, respectively), followed by stimulatory cytokine treatment (e.g., LPS/IFN-g, IL-4, and TGF-b to induce activated M1, M2a, and M2c macrophages, respectively), profiling of secreted cytokines (e.g., IL-12 and IL-10 for M1 and M2, respectively), and flow cytometry analysis of cell surface proteins (e.g., CD80 and CD206 for M1 and M2, respectively), yet these approaches do not capture the full spectrum of macrophage differentiation and adaptability. Here, we sought to use single-cell RNA-seq to explore the diversity of the innate immune response, as well as to understand its ability to be reprogrammed by treatment with an antimicrobial therapeutic. We chose the antimicrobial compound K21, a quaternary ammonium silane compound that exhibits broad-spectrum effects against viruses, bacteria, and fungi, as well as potent wound healing activity^8–13^. We found that supplementing cytokine and cell surface marker profiling with scRNA-seq transcriptomic analysis gives unparalleled resolution of macrophage diversity and plasticity, allowing us to demonstrate how treatment with K21 remodels the macrophage repertoire inside and outside the cell, and specifically within the context of surrounding immune cells and fibroblasts. We show that a key aspect of K21 treatment is the induction of mitochondrial fission and autophagy (mitophagy). We turned to the distantly related model system *C. elegans* to examine whether the mechanism used by K21 is employed *in vivo* and evolutionarily conserved. K21 induced DRP-1-mediated mitochondrial fission and mitophagy *in vivo* without impairing viability, development, or fertility; indeed, it reprogrammed metabolic gene expression and extended lifespan in these nematodes, consistent with its observed effects on mammalian cells. Taken together, our results reveal how metabolic changes in the innate immune system can be leveraged by a potent antimicrobial and sanative agent, with many of these aspects conserved from nematodes to humans.

## RESULTS

### K21 alters Monocyte-to-Macrophage Differentiation

**Figure 1:**
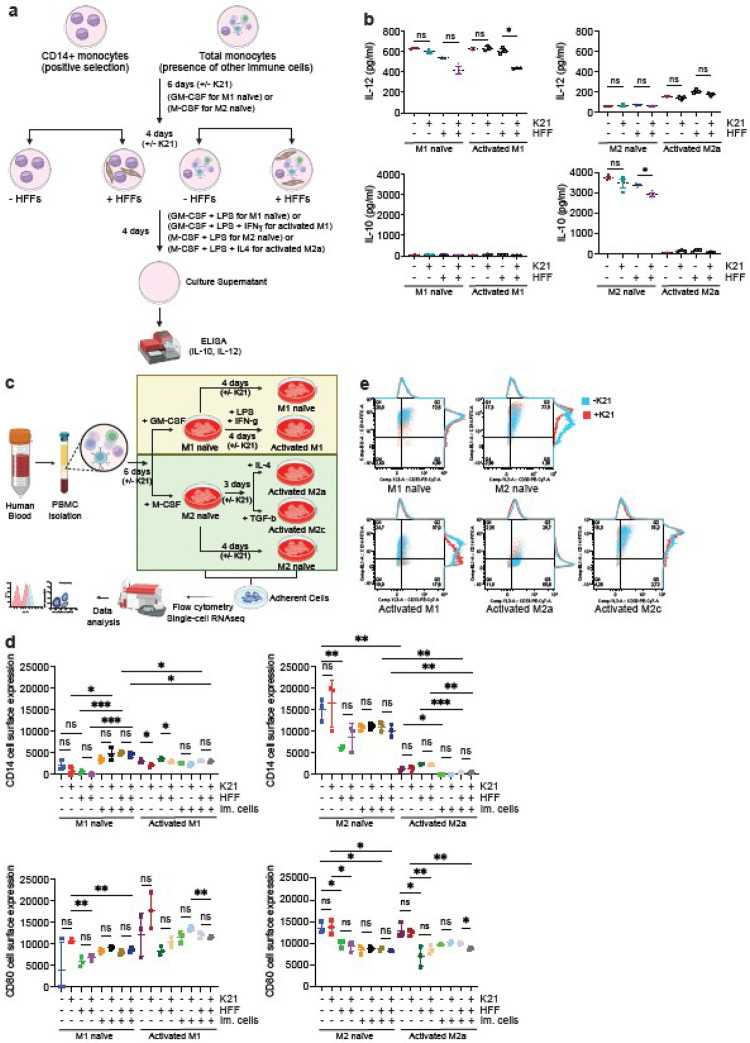
K21 Alters Cytokine Secretion and Cell Surface Protein Expression on Macrophages. (a) Schematics of the experimental setup to study the cytokine secretion profiles (IL-10 and IL-12) of macrophages by ELISA. (b) Scatter plots for secreted IL-12 and IL-10 amounts from CD14+ macrophages grown in the absence of immune cells from three independent experiments (n=3). *P<0.05, unpaired t-test with Welch’s correction. HFF, human foreskin fibroblast; ns, not significant. (c) Schematics of the experimental setup to study cell surface protein expressions and gene expression profiles of macrophages at the single-cell level. (d) Scatter plots for median fluorescent intensity (MFI) of cell surface CD14 and CD80 from macrophages from three independent experiments (n=3). *P<0.05, **P<0.005, ***P<0.005, unpaired t-test. HFF, human foreskin fibroblast; ns, not significant. (e) Dot plots for CD14 vs CD33 cell surface expression on macrophages grown in the presence of the immune cell repertoire. Representative image of one replicate experiment.

Antimicrobials primarily target specific aspects of the pathogen’s lifecycle to inhibit pathogen spread, yet few drugs can effectively control viruses, bacteria, and other pathogens, including fungal infections, while improving cellular health. The only possible way to achieve this is to alter the host immune repertoire in a way that can effectively control pathogenic survival. We used an *in vitro* primary human monocyte-derived macrophage model to test this hypothesis for the antimicrobial K21. First, we characterized various macrophage populations isolated from human blood but not yet exposed to K21 (Figure 1a) by a combined analysis of macrophage cytokine secretion profiles (IL-10 and IL-12), cell surface protein expression profiles (CD14, CD33, CD80, CD206, and CD163), and transcriptomics at single-cell levels (Extended Data Figure 1). IL-10 and IL-12 secretion patterns clearly distinguished M1 (pro-inflammatory, high IL-12 secretion) macrophages from M2 (anti-inflammatory, high IL-10 secretion) macrophages (Figure 1a and Extended Data Figure 1a). The presence of other immune cells significantly suppressed the expression of both IL-12 and IL-10 in M1 and M2 macrophages, respectively. The presence of foreskin fibroblasts also modulated IL-10 and IL-12 expression, depending on the presence or absence of other immune cells (Figure 1a and Extended Data Figure 1a). Interestingly, while naïve M2 macrophages secreted abundant IL-10, activated M2a macrophages did not show the same secretory properties, suggesting differences in cytokine secretory properties between naïve and activated macrophages. In addition to variable cytokine secretion profiles, naïve and activated macrophages also exhibited distinct patterns of cell surface protein expression. Comparison of two different growth stages of macrophages (day 6 and day 10) revealed changes in cell surface protein expression and maturation of macrophages during the later stages of growth (Extended Data Figure 1c. Single-cell transcriptomics also elegantly separated M1, M2 naïve, and activated macrophages into distinct clusters based on their individual gene expression profiles (Extended Data Figure 1d-1f). Furthermore, the extent of the monocyte-to-macrophage ratio and the state of cellular differentiation under naïve and activated conditions clearly captured the gene expression differences between these two macrophage populations (Extended Data Figure 1e-1f). These results corroborate the known knowledge about macrophages and suggest that each macrophage type carries its signature both inside and outside the cell.

We next investigated the effects of K21, which is non-toxic to human peripheral blood mononuclear cells (PBMCs) at micromolar concentrations^8^, on the monocyte-to-macrophage differentiation process based on the above three experimental approaches. K21 decreased both IL-12 and IL-10 secretion from activated M1 and M2 macrophages, respectively, but only in the presence of other immune cells and primary HFFs (Figure 1b and Extended Data Figure 2a). These results suggest that K21 slightly dampens the cytokine secretion behavior of macrophages without exhibiting a specific preference for pro- or anti-inflammatory cytokines.

Cell surface marker expression on both M1 and M2 macrophages was dependent on the presence or absence of other costimulatory immune cells and HFFs. K21 had a limited effect on the cell surface protein expression of both M1 and M2 macrophages (Figures 1c–1e). After 10 days of differentiation, naïve M2 macrophages showed maximum CD14 cell surface expression compared to other macrophages. The presence of immune cells induced CD14 cell surface expression in naïve M1 macrophages (Figure 1d). In general, activated M1 macrophages had lower cell surface CD14 expression than naïve M1 macrophages (Figures 1d–1e). Suppressed CD14 expression was also observed on activated M2a macrophages (Figures 1d–1e) but not on naïve M2 and activated M2c macrophages (Figure 1e). These effects were independent of the presence of other cell types. However, the presence of other immune cell types further decreased CD14 expression on activated M2a macrophages (Figure 1d). HFF alone decreased CD14 cell surface expression on naïve M2 macrophages (Figure 1d). K21 suppressed cell surface CD14 expression on activated M1 macrophages (Figures 1d–1e) but not on naïve M1 macrophages.

K21 had little effect on the cell surface expression of CD33, CD80, CD163, and CD206 (Figures 1d–1e, Extended Data Figures 2b-2c). The presence of both HFF and immune cells decreased CD80 expression on M1 and M2 macrophages (Figure 1d). K21 did not show any significant effect on the cell surface expression of CD206 and CD163 (Extended Data Figure 2c). The presence of HFF significantly suppressed cell surface CD33 expression in all types of macrophages (Extended Data Figure 2c). The presence of both HFFs and immune cells was necessary for the differentiation of both activated M1 and M2a macrophages. Further comparison of naïve macrophages at day 6 and day 10 revealed that K21’s effect on CD80 and CD206 cell surface expression was only measurable late in naïve macrophage differentiation (between 6 and 10 days) (Extended Data figure 2d). Based on alterations in the cell surface expression of CD14, we concluded that K21 induces M2 naïve macrophages to an M2c-like phenotype, which resembled naïve M1 phenotype but without altering the cytokine secretion properties. These results suggest that K21 does not switch the complete M1 and M2 macrophage phenotypes and has no significant effect on the cell surface protein expression of M1 and M2 macrophages. However, it remodels individual components of naïve macrophages, which might have functional significance.

### K21 Rewires Macrophage Function and Behavior

**Figure 2:**
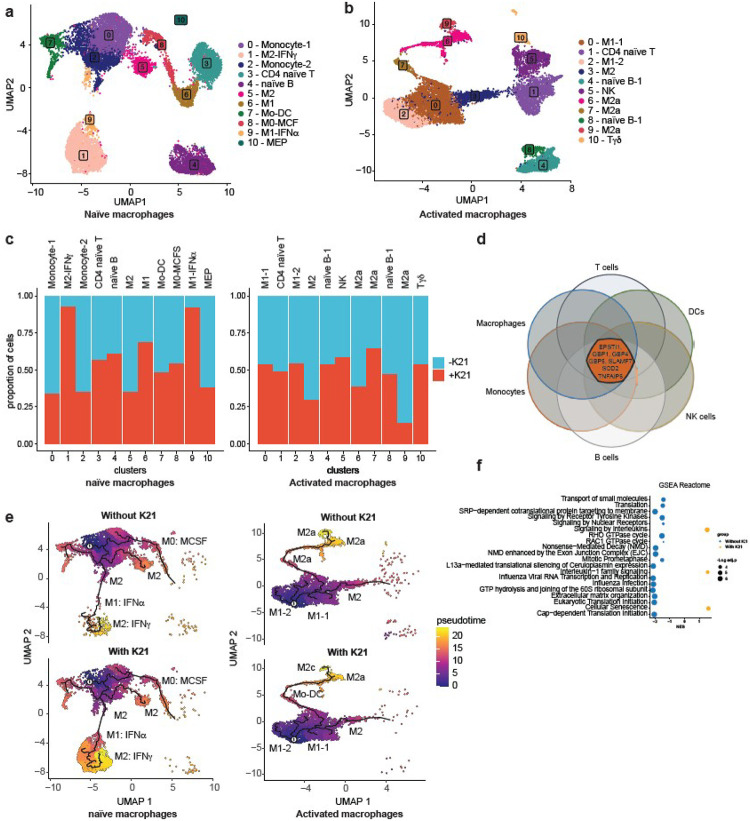
K21 Alters Gene Expression Profiles of Macrophages and Selectively Induces Growth of Pro-inflammatory Macrophages. (a) UMAP plots of 11 clusters of cells identified from single-cell RNA-seq analysis of K21-treated and untreated naïve macrophages. Cluster-specific cell types are indicated. (b) Similar UMAP analysis of activated macrophages. (c) A barplot showing changes in the proportion of various cell types (naïve on the left, activated on the right) in the presence (red) and absence (blue) of K21. (d) A Venn diagram showing the common genes upregulated by K21 in all the identified cell types. (e) UMAP visualization of trajectory and pseudotime computed from macrophage scRNA-seq data (naïve on the left, activated on the right, K21-treated on bottom, untreated on top). Cells are coloured by pseudotime. Only monocyte-derived cell populations are included within the images. (f) Bubble plot showing significantly enriched pathways from the pairwise GSEA Reactome analysis of the two pro-inflammatory macrophage populations enriched within naïve macrophages. The significantly enriched pathways are visualised for cells with and without K21. The size of each bubble is proportional to the number of core genes within the pathway. More details of the significant pathways are provided in Extended Data Figure 10. NES, Normalised Enrichment Score.

K21 did not induce complete class switching behavior of macrophages, but instead induced subtle changes in both cytokine secretion and cell surface protein expression properties. To understand the fine details of changes in the gene profile of macrophages in the presence of K21, we pooled all PBMC’s into four separate libraries based on two variables: (1) naïve versus activated (with M1 and M2 macrophage populations combined), and (2) the presence or absence of K21 (Figure 1c). We carried out single-cell transcriptomics of the immune cell repertoire after 10 days of differentiation (Figure 1c). UMAP cell clustering of the entire cell pool (naïve and activated macrophages, with and without K21 treatment) successfully identified various immune cell types (Extended Data Figure 3a). Unique gene and transcript numbers, as well as mitochondrial RNA, were comparable in all 4 sample types (Extended Data Figure 3b). Subsequent UMAP cell clustering of separated naïve and activated macrophages also efficiently captured different immune cell types in each of those two populations (Figure 2a–2b). Cell type distribution analysis recapitulated the flow cytometry results. Two distinct M1 naïve macrophage populations with the characteristics of IFNa- and IFNg-stimulated pro-inflammatory macrophages were enriched in the presence of K21 (Figure 2c, left panel; Extended Data Figure 4a, 4b). At the same time, another distinct cluster of M1 naïve macrophages with the characteristics of IFNa-stimulated macrophages was suppressed by K21 (Figure 2c, left panel; Extended Data Figure 5a). Activated M1 macrophages with the same gene expression profile were also suppressed by K21 (Figure 2c, right panel; Extended Data Figure 5b). Additionally, the number of classical M2c macrophages significantly decreased in the presence of K21.

K21 induced distinct clusters of naïve macrophage populations (Extended Data Figure 6a), which was not seen in activated macrophages. Therefore, we focused subsequent analysis on naïve macrophages only. Differential gene expression analysis of individual cell types within naïve macrophages (Extended Data Figure 6b) revealed cell-type-specific effects of K21. However, six genes (Figure 2d) were significantly upregulated by K21 in all the immune cell types. There were no common genes that were downregulated in all the cell types. To understand the transcriptional basis of K21-induced cell clusters in more detail, we analyzed differential gene expression profiles in the two distinct populations of macrophages (Extended Data Figure 7a-7b). The smaller macrophage population lacked expression of CD163L1 and hence was identified as M1 naïve macrophages and had an IFN-a stimulated macrophage profile (Extended Data Figure 7a). The largest K21-induced naïve macrophage population expressed CD163L1 and hence was identified as M2 naïve macrophages and had an IFN-g stimulated macrophage profile (Extended Data Figure 7b). Interestingly, both these macrophage populations showed very similar yet distinct pro-inflammatory gene expression profiles (Extended Data Figure 7c-7d) with enhanced expression of IDO1, IDO2 and KYNU (Extended Data Figure 7e-7f). Pseudotime trajectory analysis further corroborated the effects of K21 on macrophage polarization during the late stages of cell differentiation (Figure 2e; Extended Data Figure 8a-8b). K21-induced naïve macrophage populations appear at a distinct and late time point of differentiation. M2a and M2c macrophages also differentiate on a distinct trajectory than M1 macrophages. Gene Set Enrichment Analysis (GSEA) revealed uniform enrichment for Interleukin signalling, IL-1 signalling, and cellular senescence in the presence of K21, which was particularly prominent in pro-inflammatory macrophage populations (Figure 2f; Extended Data Figure 9b-9e). Gene Ontology (GO) biological processes analysis revealed enhanced responses in macrophages to LPS, bacterium, viral infections, and other inorganic and biotic stimulus in the presence of K21 (Extended Data Figure 9a). These systems biology approaches suggested that K21 induces distinct pro-inflammatory macrophage populations out of both M1 and M2 macrophages that enhance the ability of the macrophages to fight infections. Additionally, the induction of specific genes in the presence of K21 suggests metabolic reprogramming that makes macrophages more powerful in preventing various types of infections.

### K21 Induces Mitophagy and Remodels Mitochondrial Metabolism

**Figure 3:**
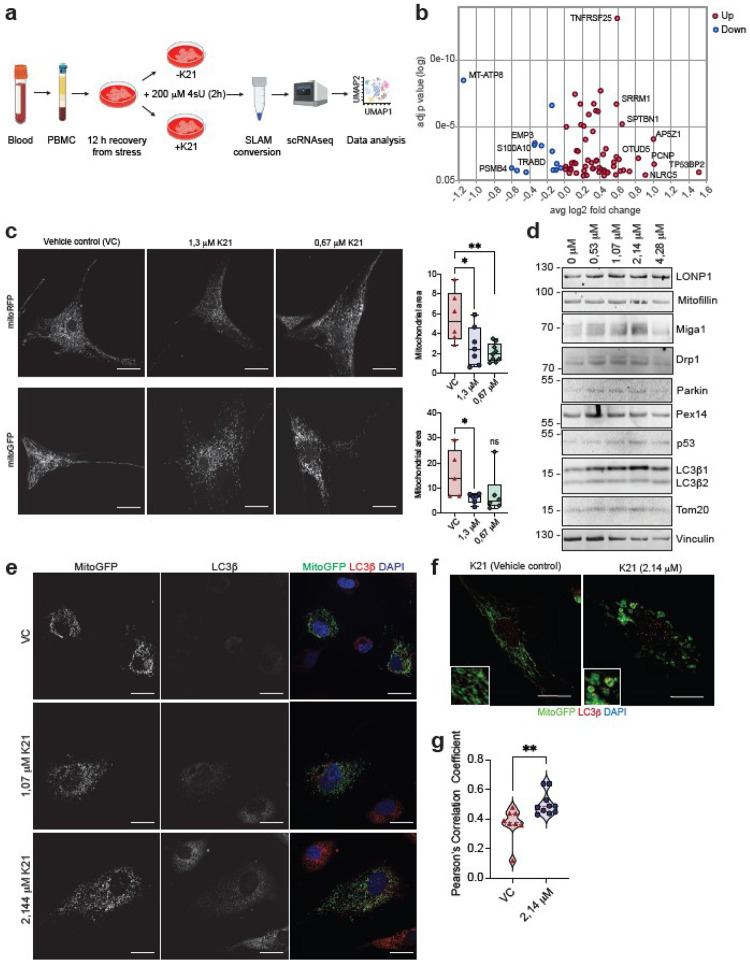
K21 Favors Mitochondrial Fragmentation and Mitophagy. (a) Schematics of the experimental setup to study dynamic changes in RNA expression within the first two hours of K21 exposure in peripheral blood mononuclear cells (PBMCs). (b) Bubble plot of log_2_ fold changes and log_10_ adjusted P values for individual newly synthesized genes with log2FC > 0.5 and adjusted P value < 0.05 as detected by scRNA-seq in the presence of K21. Top candidate genes upregulated (red) or downregulated (blue) are indicated. (c) Visualization of K21-induced mitochondrial fragmentation in HFF cells expressing stable mitoRFP (upper panel) or mitoGFP (lower panel) using confocal imaging. Bar, 10 microns. Average mitochondrial surface area was quantified from at least five independent images per condition and is plotted as a Box and Whiskers plot. *P<0.05, **P<0.005, unpaired Mann-Whitney test. VC, vehicle control; ns, not significant. (d) Immunoblots showing concentration-dependent changes in protein expression in the presence of K21 in HUVEC cells. Original uncut blots are shown in Supplementary Figure 2. (e) Confocal images from U2-OS cells expressing stable mitoGFP, showing a concentration-dependent increase in LC3b expression. (f) Confocal images of HUVEC cells showing colocalization of LC3b to mitochondrial fission compartments. (g) Violin plot showing Pearson’s correlation coefficient from at least 10 independent images. (n=10). **P<0.005, unpaired Mann-Whitney test. VC, vehicle control.

Out of the six core genes induced by K21 (Figure 2d), GTPase binding protein GBP1 induces mitochondrial fission and enhances macrophage health. SOD2 improves mitochondrial health through its ROS-scavenging functions. GBP5 is a known interferon-inducible antiviral protein. As reprogramming mitochondrial health and metabolism is integral to immune responses^7,14,15^, we asked if K21 directly targets mitochondria. We utilized a single-cell SLAM-seq approach in human PBMC to study alterations in gene expression within the first two hours of K21 exposure (Figure 3a). SLAM-seq pulse-labels newly synthesized RNA within a specific time frame and hence allows for the precise identification of transcription rates of genes in response to a particular stimulus (Extended Data Figures 10a-10b). Only T and NK cells were metabolically active to capture enough 4sU incorporation in these cells within 2 hours of culture. Monocytes and B cells were less in number and did not incorporate enough 4sU within the 2 hours of the experiment (Extended Data Figure 10c) to enable effects of K21 on these cell types. Both GSEA Reactome and GO-BP analysis (Extended Data Figures 10d-10e) showed altered type I interferon response, biological oxidation in T cells, and enhanced interleukin response in NK cells in the presence of K21. We identified significant suppression of transcription of genes (Mt-ATP8, TRABD) associated with mitochondrial function and calcium signalling (S100A10) in response to K21 treatment only in T cells (Figure 3b). The suppression of Mt-ATP8 was relevant as our scRNA-seq data also identified a specific cluster of macrophages enriched for Mt-ATP8 that was depleted in the presence of K21 (Extended Data Figure 5a). This suggests that K21 possibly affects mitochondrial function in all treated cells given enough time.

As TRABD enhances mitochondrial fusion, we reasoned that a K21-induced decrease in TRABD transcription should facilitate mitochondrial fission. We tested the effects of K21 on mitochondrial architecture in primary human foreskin fibroblasts (HFFs), primary human umbilical vein endothelial cells (HUVECs), and U2-OS cells, all of which showed induction of strong mitochondrial fragmentation in the presence of K21, as measured by a decrease in average mitochondrial surface area (Figure 3c). We observed increased LC3b expression in the presence of K21 (Figure 3d–3e) along with an increase in Drp1, Miga1, and LONP1, suggesting K21 induced mitophagy. This result was further validated by LC3b-specific staining in HUVECs, where both mitochondria and LC3b colocalized with a significant Pearson’s Correlation Coefficient (p=0.0025). An increase in Pex14 also suggested the induction of peroxisome biogenesis in the presence of K21. These observations corroborated scRNA-seq data from macrophages, suggesting that K21-mediated mitochondrial recycling pathways enhance mitochondrial health and metabolism.

### K21 Induces Mitophagy and Lifespan Extension in *C. elegans*

To determine whether K21 can induce mitophagy and remodel metabolism more broadly, we turned to the *in vivo* model system *C. elegans*. Mitophagy can be observed in these nematodes using transgenes expressing MitoKeima, a protease-resistant, pH-sensitive fluorescent protein localized to the mitochondrial matrix that is used to monitor mitophagy through dual-excitation fluorescent imaging^16,17^ (Extended Data Figure 11). We generated a transgene, *odIs167*, that expresses MitoKeima in the *C. elegans* intestine. Intestinal MitoKeima was detectable in the mitochondrial network with 470 nm excitation (Extended Data Figure 12); these labeled networks could be decorated with Mitotracker green staining (Extended Data Figure 13). Known inducers of mitophagy, including starvation and CCCP treatment, created a second population of MitoKeima-labeled spherical structures detectable with 555 nm excitation (Extended Data Figure 12); these structures could be decorated with Lysotracker green staining (Extended Data Figure 13). Formation of these acidified mitophagosomes could be blocked by mutations in *lgg-1*, which encodes a *C. elegans* homolog of the autophagy adaptor LC3 essential for mitophagy (Extended Data Figure 14), demonstrating MitoKeima as a faithful *in vivo* mitophagy reporter for the *C. elegans* intestine.

**Figure 4:**
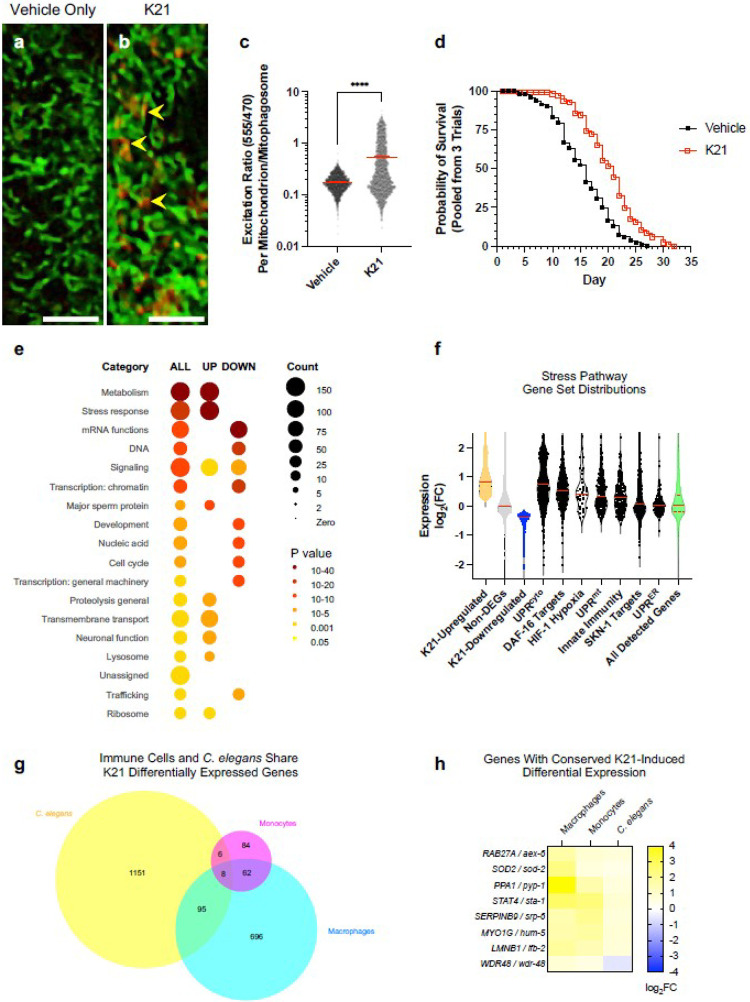
K21 Induces Mitophagy And Lifespan Extension In *C. elegans*. (a,b) Dual channel image of intestinal MitoKeima fluorescence combining emission from 470 nm excitation (non-mitophagic mitochondria, green) and 555 nm excitation (mitophagic mitochondria, red) in (a) vehicle-treated or (b) K21-treated L4 nematodes. Arrowheads point to example mitochondria undergoing mitophagy. Bar, 5 microns. (c) Ratio of fluorescence from excitation of 555 nm relative to that at 470 nm for individual mitochondria from 5 vehicle-treated versus 5 K21-treated animals. ****P<0.0001, t-test. Mean indicated by red bar. (d) Kaplan-Meier survival curves for vehicle-treated (black circles) or K21-treated (red squares) animals, pooled from three independent trials (n=128 and 151 animals, respectively). P<0.0001, Mantel-Cox log-rank. (e) WormCat category enrichment for either all DEGs induced by K21 or DEGs either up- or down-regulated by K21 vs vehicle, as indicated by the column headers. Circle size and shading represent number of genes identified in the category and the p-value for that category (adjusted for multiple comparisons), respectively, as per the legend. (f) Violin plots of mRNA expression levels (as log2 fold change) for individual genes for K21-treated versus vehicle-treated nematodes, separated into significantly (FDR<0.01) upregulated genes (gold), downregulated genes (blue), genes showing no significant differential regulation (gray), and all detectable genes combined (green). Other columns show the distribution of expression values within the RNA-seq for genes upregulated within the indicated stress response pathway gene set. Red solid line indicates the mean. (g) Venn diagram showing overlap between differentially expressed genes for K21 versus vehicle treatment from macrophage scRNA-seq (cyan), monocyte scRNA-seq (magenta), and *C. elegans* RNA-seq (yellow). Only genes showing orthology between humans and *C. elegans*, as well as differential expression in one or more datasets, are shown. (h) Hierarchically clustered heat map of log2 fold changes in mRNA levels from K21 versus vehicle-treated samples (as labeled in the columns) for only orthologous genes, and only for those showing differential expression in all three samples: macrophages, monocytes, and *C. elegans*.

To deliver K21 to *C. elegans*, we generated a colloidal suspension of micron-sized K21 particles and layered these over a bacterial OP50 lawn. Nematode eggs were hatched on this OP50/K21 mixture and allowed to feed and grow to the L4 stage, when they were assessed for MitoKeima fluorescence. K21-treated nematodes showed increased DRP-1-dependent fission (Extended Data Figure 15) and mitophagy compared to untreated controls (Figure 4a–c). The K21 suspension did not kill the OP50 food source or impair the ability of nematodes to feed (Extended Data Figure 16). Treatment did not impair survival, development, growth, or fertility (Extended Data Figure 17). Indeed, K21 extended the lifespan of treated nematodes (Figure 4d). Mitophagy can be induced by mitochondrial stress^18,19^, and stress-induced activation of the mitochondrial unfolded protein response (UPR^mt^) extends lifespan in *C. elegans* mutants with impaired mitochondrial function^20^. However, we did not observe activation by K21 of the established UPR^mt^ reporter *hsp-6::gfp*, and mutants for *atfs-1*, the key activator of the UPR^mt 21^, still showed lifespan extension following K21 treatment (Extended Data Figure 18), suggesting K21 does not induce mitophagy or lifespan extension by inducing mitochondrial stress.

To understand how K21 might be affecting *C. elegans*, we used RNA-seq to obtain a transcriptional profile of K21-treated nematodes. Principle Component Analysis (PCA) showed tight clustering across replicates, and we identified 1848 differentially expressed genes (DEGs) upregulated by K21 and 1312 DEGs downregulated by K21 using a false discovery rate (FDR) ≤ 0.01 (Extended Data Figure 19; Supplemental Data 1). Gene Ontology (GO term) analysis using either WormCat (Figure 4f) or gProfiler (Extended Data Figure 20) showed that K21 induced the expression of genes involved in lipid metabolism, the TCA cycle, OXPHOS/ETC, stress response, proteolysis, the endolysosomal system, and general mitochondrial and peroxisomal function. K21 reduced the expression of genes associated with DNA replication, the cell cycle, transcription, mRNA processing, and translation.

The WormCat enrichment for stress response genes led us to analyze individual stress response pathways. We used Gene Set Enrichment Analysis (GSEA) to examine the distribution of gene sets known to be upregulated by various stress response pathways^22^ within the K21 RNA-seq data. Genes upregulated by the heat shock response pathway (UPR^cyto^), DAF-16, HIF-1, the UPR^mt^, SKN-1, and innate immune response showed a distribution similar to that of all K21-upregulated genes (Figure 4f), and GSEA analysis showed significant enrichment for these gene sets in K21-upregulated genes (Extended Data Figure 21). Although the UPR^mt^ gene set was enriched in the full set of K21-upregulated genes, *hsp-6* expression did not show a statistically significant upregulation by K21 in our RNA-seq data, matching our *hsp-6::gfp* analysis. Surprisingly, a gene set for dietary restriction (i.e., upregulated in *eat-2* mutants compared to wild type^23^ was enriched in the K21-upregulated genes despite K21-treated animals showing similar feeding compared to untreated animals (Figure 4f), suggesting that K21 can mimic dietary restriction. Taken together, these results show that K21 treatment can partially activate the expression of a large array of stress response pathway targets.

Finally, we reasoned that there might be a conserved core of K21-regulated genes conserved across evolution. We examined common K21-induced DEGs from the scRNA-seq analysis of monocytes and macrophages, which comprised the two largest cellular populations. We then used OrthoList2 to identify potential *C. elegans* orthologs of these DEGs within the nematode RNA-seq^24^. Nematode orthologs showing K21-induced differential expression could be found for many orthologous DEGs induced by K21 in monocytes or macrophages (Figure 4g), and for 8 genes showing differential regulation in both monocytes and macrophages, we could observe differential regulation in their *C. elegans* orthologs (Figure 4h). Taken together, these results demonstrate that K21 remodels metabolism, activates stress response pathways, and promotes mitophagy in *C. elegans*, with many of these aspects conserved from nematodes to humans.

## DISCUSSION

Antimicrobial compounds demonstrating broad-spectrum effects against viral, bacterial, and fungal pathogens typically achieve these effects through surfactant properties and other physical and chemical mechanisms, including the solubilization of lipid membranes and the denaturation of proteins. Such agents at high concentrations make for excellent disinfectants and topical treatments when formulations are carefully chosen to be non-irritating to human skin, but can have limited utility to treat infections that have breached barrier tissues (e.g., wounds) and/or formed microbial biofilms, or to treat internal infections^25,26^. A small number of compounds, including the quaternary ammonium silane compound K21, demonstrate broad-spectrum effects even at low concentrations, with minimal toxicity to human cells. Here, we provide evidence that K21 achieves its broad-spectrum effectiveness by modulating the innate immune system. Using a powerful combination of cytokine profiling, cell surface marker flow cytometry, and scRNA-seq to describe the granular spectrum of macrophage differentiation and plasticity, we find that K21 promotes the differentiation of pro-inflammatory macrophages from M1 and M2c populations without changing their plasticity and ability to secrete their respective cytokines. K21 also decreased specific subtypes of M1 macrophages and CXCL4-induced M2-like macrophages (i.e., M4 macrophages). The combined effects of these changes likely explain the ability of K21 to combat a broad array of pathogens and accelerate wound healing^8–13^.

K21 significantly induced two clusters of macrophages characterized by their strong pro-inflammatory and pathogen-clearance properties. These two cell clusters (cluster 1 and cluster 9, Figure 2c) were not detected in activated macrophages, regardless of whether K21 was present or not, and they also did not cluster when non-K21 samples were compared (Extended Data Figure 1d–1e). Cells from cluster 9 resembled typical IFNa-induced M1 macrophages. They lacked expression of M2 markers, such as CD163L1 and PPARd^27,28^, and exhibited very low expression of other M2 macrophage markers, including FMN1 and DAPK1. However, activated M1-specific markers, such as CCL-5 and CD38, were strongly induced in the presence of K21 in these cells. From these observations, we concluded that these cells are M1 macrophages, which were modulated to another subtype of M1 cells capable of a better pro-inflammatory response. At the same time, cluster 1 was unique and resembled M-CSF-IFNg-Pam3Cy5 cells from SingleR annotation. M2-specific markers, such as CD163L1 and PPARd, were detectable in these cells and were further suppressed by K21. These cells also had very strong expression of M2 macrophage markers, including FMN1 and DAPK1. Activated M1-specific markers, such as CCL-5 and CD38, were strongly induced in the presence of K21 in these cells. K21 further induced M1-specific proteins like CXCL8 and CXCL9 in this cluster of cells^29^. These clusters of cells also induced IL-17 expression, which is essential for M1 differentiation. From these observations, we concluded that these cells were originally M2 macrophages that had been switched towards an M1-like phenotype in the presence of K21. These supercharged macrophages exhibit both pro-inflammatory and anti-inflammatory properties simultaneously. Both of these clusters were characterized by K21-induced expression of proteins like SLC1A2, WARS1, SLAMF7, KYNU, SNX10, and CHI3L1, which are critical for initiating and amplifying a pro-inflammatory response necessary for pathogen clearance and recruitment of other healing cell types. SLAMF7, in particular, is known to superactivate macrophages under chronic and inflammatory conditions^30^.

K21 induces the expression of kynureninase (KYNU), IDO1, and IDO2, key enzymes of the tryptophan-kynurenine pathway. IDO1 and IDO2 catalyze the cleavage of tryptophan to kynurenine, which is subsequently converted to anthranilic acid by KYNU. Tryptophan is an essential amino acid for the synthesis of serotonin, a neurotransmitter that plays a crucial role in regulating mood, sleep, appetite, and other functions. Tryptophan is also utilized for the synthesis of nicotinamide adenine dinucleotide (NAD^+^), a critical molecule in cellular energy metabolism. As another intermediate in the kynurenine metabolism of tryptophan, 3-hydroxykynurenine is converted to 3-hydroxyanthranilic acid by KYNU, leading to NAD^+^ generation. A delicate balance between these two pathways (tryptophan-serotonin versus tryptophan-kynurenine) is essential for human life.

The kynurenine pathway of tryptophan degradation is involved in T lymphocyte differentiation, influencing immune tolerance and inflammation^31^. The IFNγ-IDO1-kynureine pathway plays a critical role in inducing autophagy in cervical cancer cells and promotes phagocytosis by macrophages^32^. Kynurenine is a stable intermediate of the kynurenine pathway, and increased levels (along with increased IDO1 and IDO2) are often detected within the tumor microenvironment, suggesting their negative impact on tumor growth^33^. However, overexpression of KYNU should resolve this negative aspect of the kynurenine pathway by efficiently salvaging kynurenine. We did not detect KYNU within untreated macrophages. However, KYNU was strongly induced within the two pro-inflammatory clusters of macrophages induced by K21. Increased IDO1 and IDO2 are indirectly linked to reduced serotonin levels, depression, and mood disorders. At the same time, the positive role of IDO1 and IDO2 in immune system development is also documented^34,35^. We observed increased IDO1 expression in T cells, B cells, monocytes, and macrophages in the presence of K21. However, IDO2 induction was observed only in macrophages along with the induction of KYNU. These results suggest a specific positive role of the kynurenine pathway in the pro-inflammatory pathogen clearance function of macrophages. These arguments could be tested in future studies using an animal model where serotonin levels could be measured after treatment with K21.

We detected a cluster of macrophages under both naïve and activated conditions, which showed high expression of LY6E, MMP9, SPP1, MT-ATP8, MT-ND3, MT-ND6, and actin B. These M2-like macrophages, characterized by high MMP9 expression^29^, were also identified under normal macrophage differentiation conditions without the influence of K21. However, K21-treated cells suppressed this cluster of macrophages. These macrophages resemble monocyte-derived Tumor-Associated Macrophages (TAMs), often found within the tumor microenvironment. TAMs significantly contribute to cancer progression and are active targets for cancer immunotherapy. Our SLAM-seq approach showed a significant decrease in MT-ATP8 RNA levels in response to K21 treatment, suggesting inhibitory effects of K21 on MT-ATP8 expression. Our pseudotime trajectory analysis showed independent developmental branching of these M2-like macrophages from other M2 macrophages. Based on this analysis in naïve macrophages (Figure 2e), we speculate that K21 potentially alters the plasticity of these macrophages to an M1 phenotype. Suppression of TAM-like macrophages through K21 provides a novel drug-induced approach to control tumor progression. Mulder et al. have identified unique TAMs (IL4I1^+^ CD274 (PD-L1)^+^ IDO1^+^), which accumulate in the tumor periphery in a T cell-dependent manner via interferon-γ (IFN-γ) and CD40/CD40L-induced maturation from IFN-primed monocytes^36^. In this study, we demonstrate an increase in K21-induced M2-primed IFN-γ-stimulated IDO1^+^ macrophages, characterized by decreased CD274 expression and absence of IL4L1 and CD40/CD40Lexpression. These minor variations within macrophages could suggest significant alterations in their properties. Two clusters of naïve macrophages (clusters 0 and 2), which were identified as CXCL4 monocytes by SingleR annotation, resembled M4 macrophages and were suppressed in the presence of K21. M4 macrophages are a recently identified macrophage subset^37^ with high expression of chemokine CXCL4, and they are known for their distinct characteristics and roles in various inflammatory and fibrotic processes. They are considered atherogenic due to their production of pro-inflammatory cytokines and reduced phagocytic capacity^38^. Using scRNA-seq and spatial transcriptomics, Coulis et al. have recently identified a novel population of macrophages with high expression of galectin-3 and SPP1 in skeletal muscle fibrosis^39^. Our data showing suppression of these macrophages with high SPP1 and MMP9 expression by K21 could be beneficial in reducing fibrosis and sclerosis.

Metabolic differences, including the regulated balance between glycolysis, OXPHOS activity, and lipid metabolism, are critical drivers of M1 versus M2 polarization. Mitochondria lie at the fulcrum of this balance, and interestingly we found that K21 treatment induces mitochondrial fission and mitophagy, as well as regulates the expression of multiple genes involved in mitochondrial metabolism, morphology, and quality control, suggesting mitochondria could be the mechanistic target of this agent. M1 macrophages rely on glycolysis, the pentose phosphate pathway, and fatty acid synthesis, whereas M2 macrophage metabolism is characterized by elevated fatty acid oxidation and OXPHOS activity^4–7^; thus, the ability of K21 to modulate mitochondrial metabolism, dynamics, and quality control could explain how this agent promotes the antimicrobial abilities of these cells without dramatically altering their cytokine secretion profiles. Aside from energy and ATP production, differences in macrophage metabolism along the M1/M2 polarization axis also impact the ability of these cells to produce metabolites and the small molecule signals used to fight infections and communicate between innate and adaptive immune cells. Indeed, K21-mediated immune cell remodeling was observed only in a complex multicellular co-culture environment, with T cells and NK cells rather than macrophages being the first cells to show gene expression changes in our SLAM-seq analysis, suggesting an interplay of various cell-derived components is required for K21 to act. The example of how K21 can modulate innate immunity below the surface of cytokine secretion and cell surface marker expression highlights the need to understand the differentiation and plasticity of innate immune cells using detailed and granular systems approaches, including single-cell RNAseq and transcriptomics.

Interestingly, the effects of K21 on mitochondrial fission and mitophagy were observed in the model organism *C. elegans*, demonstrating an *in vivo* context shared by our observation of the effects of K21 on PBMCs in culture. Nematodes do not possess macrophages or other well-established components of human innate and adaptive immunity; nevertheless, genes associated with innate immunity in *C. elegans* were upregulated by K21 treatment. Although the effects of K21 on immunity may not be analogous to those in humans, we found that K21 treatment extended thelifespan of *C. elegans* without compromising survival or fertility, consistent with increased activity of mitophagy as a quality control mechanism that boosts health through hormesis^40,41^. Interestingly, genes upregulated by dietary restriction (i.e., upregulated in *eat-2* mutants, which are impaired for feeding) were upregulated by K21 treatment even though such treatment had no effect on feeding compared to eat-2 controls, indicating that K21 could be mimicking dietary restriction without reducing food intake, which could be contributing to its extension of lifespan. As nematodes and humans are separated by nearly a billion years of evolution, the overall conserved effects of K21 suggest that the molecular or pharmacological target of K21 is deeply conserved and not specific to immune cells. The mitochondrial organelle itself is fundamentally conserved and plays critical roles in metabolism, immunity, and lifespan. An understanding of how K21 acts on or through mitochondria should facilitate the development of additional powerful therapeutics in the future.

## ONLINE METHODS

### Macrophage Differentiation and Flow Cytometry

Macrophages were generated from human peripheral blood mononuclear cells (PBMCs) of healthy donors. Two different modes of peripheral blood mononuclear cells (PBMCs) collection were used. For the initial comparison of the effects of immune cells and fibroblasts on macrophage differentiation, PBMCs were collected from EDTA blood of healthy blood donors. Peripheral blood for these studies was obtained from healthy adult donors, with ethical approval from the Riga Stradiņš University (RSU) Research Ethics Committee (2-PĒK-4/784/2025). After 10 days of differentiation, the culture media were removed from the wells. Cells were rinsed with PBS to remove residual media. 500 mL ice-cold EDTA (10 mM) was added to each well and incubated at 4 °C for 30 min to 1 hour. Cells were pipetted to detach from the surface and were transferred to a 96-well plate. Cells were centrifuged at 550 g for 5 minutes to remove the debris. Cells were then blocked with 50 μL Fc block (1:200) at 4 °C for 30 minutes. Cells were then centrifuged at 550 g for 5 minutes to remove the blocking solutions and washed with 200 μL of FACS buffer. 50 μL of antibody cocktail was added to each well and incubated at 4 °C for 30 minutes. Afterwards, cells were washed twice with 200 μL of FACS buffer and resuspended in 200 μL of FACS buffer. Protein expression was measured using BD FACS Aria III. The following antibodies were used for flow cytometry.

**Table:** 

Marker	Channel	Dillution	Cat Number	Lot	Clone
CD163	BV421	1:100	333611	B425652	GHI/61
CD80	BV650	1:100	305227	B436598	2D10
CD33	PE/Cy7	1:300	366617	B417457	P67.6
CD206	AlexaFluor 647	1:200	321116	B410991	15-2
CD14	APC/Cy7	1:200	325619	B363390	HCD14
CD11b	BV510	1:100	301334	B449230	ICRF44
Fc Block (1:200)		1:200	130-059-901	5241102824	

For subsequent Flow cytometry and single-cell RNA-seq work, heterogeneous donor-derived PBMCs were used. PBMCs were prepared from healthy blood donors as a byproduct of platelet concentrates obtained using leukoreduction system chambers, kindly provided anonymously by the Department of Transfusion Medicine at the University Hospital Würzburg, under the guidelines of the Ethics Committee of the Medical Faculty of the University of Würzburg. PMBCs were isolated using a density gradient (Histopaque-1077, Sigma-Aldrich, #1077–100ml), washed twice with PBS, and resuspended in Monocyte Attachment Medium (PromoCell #C-28051). The frequency of mononuclear cells was analyzed by Flow cytometry (Attune Nxt Flow Cytometer). As the frequency of mononuclear cells was beyond 25% of the total, 1,5 × 10^6^ cells/cm^2^ were seeded into 6-well plates (1,4 × 10^7^ per well) and were incubated for 1–2 hours at 5% CO2 and 37 °C in the incubator for cell attachment. Adherent cells were washed thrice intensively with warm Monocyte Attachment media to loosen non-adherent cells.

M1 or M2 Macrophages were generated using M1 or M2 Generation Medium XF, respectively (PromoCell M1 #C-28055 containing GM-CSF or M2 #C-28056 containing M-CSF). One group was treated with 0.2 mM K21, and the control group received vehicle control. K21 was kindly provided by FiteBac LLc., USA. Incubation at 37 °C for 6 days yielded immature macrophages. On day 6, M1 immature macrophages were activated into classical M1 macrophages by adding 50 ng/ mL IFN-γ (Immunotools rh-IFNγ #11343534) and 10 ng/mL LPS (Sigma-Aldrich, Lipopolysaccharides from *Escherichia coli* O111:B4 #L5293), or they remained without an activation stimulus. M2 immature macrophages were activated into M2a macrophages by adding 20 ng/ mL IL-4 (rh IL-4 Sigma-Aldrich #SRP3093) or into M2c macrophages by adding 20 ng/ mL TGFβ (R&D rhTGFβ #7754-BH/CF), respectively. The cells were further incubated until day 10 in the presence of K21 or without it as a control. On day 10, macrophages were washed with PBS and detached in the presence of 1 mL Accumax solution (Sigma-Aldrich #SLCR6580) for 5–10 min at 37 °C.

The resulting macrophages were analyzed for successful M1 and M2 differentiation, as well as the effect of K21 on the resulting subset frequencies, using flow cytometry. Additionally, macrophages from one of the replicates were processed for single-cell RNA sequencing analysis.

For flow cytometry analysis, all macrophages were first blocked using the blocking antibody 2.4G2 for 15 minutes at 4 °C. Next, macrophages were labeled with saturating amounts of human antibodies in FACS buffer (PBS containing 0.1% BSA and 0.02% NaN3) for 15 minutes at 4 °C. The following antibodies were used against the cell-surface antigens: CD66b, CD11b, CD14, CD16, CD163, CD80, CD206, CD33, CD11c, CD1c, CD209. Stained macrophages were directly measured using the Attune Nxt Flow Cytometer (Thermofisher). The following antibodies were used for flow cytometry. The gating strategy for flow cytometry is shown in Supplementary Figure 1.

**Table:** 

Antibody	Distributor	Cat number	clone
CD66b A700	Biolegend	305114	G10F5
CD11b BV510	Biolegend	301334	ICRF44
CD14 FITC	R&D	FAB3832F-100	FAB3832F
CD16 BV605	Biolegend	302040	3G8
CD163 PerCp	R&D	FAB1607C	215927
CD80 PE	R&D	FAB140P-100	37711
CD206 APC	R&D	FAB25342A	685641
CD33 PE-Cy7	Biolegend	303434	WM53
CD11c APC-Cy7	Biolegend	337218	Bu15
CD1c PE-Dazzle 594	Biolegend	331532	L161
CD209 BV421	Biolegend	330118	9E9A8

### Single-cell RNA-seq and single-cell SLAM-seq

For single-cell RNA-seq analysis, 1 million cells per condition were washed with PBS on day 10 of the experiment and then detached in the presence of 1 mL Accumax solution (Sigma-Aldrich #SLCR6580) for 5–10 min at 37 °C. Cells were washed once again with PBS, fixed in a PBS: methanol mix (ratio of 1:4), and stored in a −80 freezer until subsequent use. On the day of the experiment, methanol-fixed cells were thawed on ice and rehydrated in 1X PBS for 30 minutes on ice, then filtered with a 40 μm cell strainer. Cells were barcoded using MULTI-seq Lipid Modified Oligos (Sigma-Aldrich #LMO001). After cell counting, single-cell suspension samples were pooled into four separate groups (Activated macrophages, −K21; Activated macrophages, +K21; naïve macrophages, −K21; and naïve macrophages, +K21). Ten thousand cells per sample were loaded on a Chromium Controller (10× Genomics). The scRNA-seq libraries were constructed using the Chromium Single Cell 3’ v3.1 Reagent Kit according to the manufacturer’s guidelines (10× Genomics). cDNA libraries were uniquely sample-indexed and pooled for sequencing. A HiSeq (Illumina) sequencing run was used to sample balance on a NovaSeq 6000 SP flowcell (Illumina) targeting > 25,000 reads/cell.

For single-cell SLAM-seq, freshly isolated PBMCs were grown for 2 hrs in the presence (0.2 mM K21) or absence of K21, along with 200 mM 4sU added to the cell culture media at the time of K21 addition, which allowed two hours of 4sU incorporation into the cells. PBMCs were washed and fixed in methanol as mentioned above. The methanol fixation was allowed to proceed for 30 minutes at −20 °C. Then, 10 mM iodoacetamide (IAA) was added to each of the methanol-fixed cells, and the mixture was left at 4 °C overnight with gentle agitation. The next day, cells were centrifuged down and resuspended in quenching buffer containing DTT (DBPS, 0.1% BSA, 1 U/μl RNaseOUT, 100 mM DTT) for 5 min at room temperature. Cells were then centrifuged down and resuspended in resuspension buffer (DBPS, 0.01% BSA, 0,5 U/μl RNaseOUT, 1mM DTT). Cells were then passed through a 0,4m filter, counted, and analyzed by 10X Genomics microfluidics. PBMCs with and without K21 were loaded into two separate lanes on the same chip, targeting 5000 cells per lane. The scRNA-seq libraries were constructed using the Chromium Single Cell 3’ v3.1 Reagent Kit according to the manufacturer’s guidelines (10× Genomics). cDNA libraries were uniquely sample-indexed and pooled for sequencing. A HiSeq (Illumina) sequencing run was used to sample balance on a NovaSeq 6000 SP flowcell (Illumina) targeting > 25,000 reads/cell.

### Data Processing and Visualization

Experimental data were demultiplexed using the Cell Ranger Single-cell Software Suite, mkfastq command wrapped around Illumina’s bcl2fastq. Cell Ranger software was used to perform demultiplexing, alignment, filtering, barcode counting, UMI counting, and gene expression estimation for each sample according to the 10× Genomics documentation (https://support.10xgenomics.com/single-cell-gene-expression/software/pipelines/latest/what-is-cell-ranger). The gene expression estimates from each sample were aggregated using Cellranger (cellranger aggr) to compare experimental groups with normalized sequencing depth and expression data.

The sequencing data was processed using the CellRanger (v8.0.0) GEX+ADT pipeline, which conducts QC, cell filtration, read alignment, and generation of feature-barcode matrices. The reads were aligned to the human (GRCh38) reference genome. To obtain higher numbers of usable cells, we set the parameter ‘--expect-cells=10000’ within CellRanger, which resulted in slightly higher numbers of cells: 5472, 5315, 6303, and 7928 cells, respectively. Seurat (v4.1, https://satijalab.org/seurat/) R packages were used to calculate QC matrices and to filter cells and features. For filtering of cells (https://github.com/satijalab/seurat/issues/3396), we first removed empty droplets. Subsequently, we also removed low-quality cells with unique feature counts greater than 8000 or less than 200 (nFeature_RNA more than 200, nFeature_RNA less than 8000). Furthermore, low-quality or dying cells that exhibited extensive mitochondrial contamination (percent.mt > 20) were removed. Genes with expression for at least three cells were retained for filtering^42^. Quality control (QC) was based on (1) nFeature_RNA: number of unique genes detected in each cell; (2) nCount_RNA: total number of molecules detected within a cell; (3) percent.mt: percentage of reads that map to the mitochondrial genome. Mitochondrial QC metrics were generated, calculating the percentage of counts originating from a set of features (the set of all genes starting with “MT-” mitochondrial genes).

Demultiplexing was carried out to identify the 8-base Multiseq barcodes and assign reads to assumed Macrophage subtypes, e.g., M1, M2a, etc. Seurat (v4.1) R package was used to process and analyze the data. We first analyzed the data for each of the four samples separately, focusing on the demultiplexing outcome. The feature expression count for each cell was normalized by the total count. The normalized count for each cell was multiplied by a scale factor (10000), then log-transformed. The expression measure was then adjusted to account for variation in mitochondrial gene proportions. For the identification of highly variable features, the 2000 most variable features were selected. Afterward, cell clustering and Principal Component Analysis (PCA) were performed. Uniform Manifold Approximation and Projection (UMAP) and clustering were performed to identify clusters. Graph-based cell clustering was performed. The PBMC SLAM-seq data were processed using the GrandR package, developed by the Erhard group^43^.

### Gene Set Enrichment Analysis (GSEA) for scRNA-seq data

GSEA analysis for each major annotated cell type was carried out using GO database, including Biological Process (BP), Cellular Component (CC) and Molecular Function (MF). GSEA Reactome analysis was carried out using C2 signature dataset from MSigDB. Differentially expressed genes were identified using the FindMarkers() function in Seurat with parameters logfc.threshold=0 and return.thresh=1. The log2-fold change calculation from FindMarkers() was used for GSEA. GSEA was performed using the fgsea (version 1.30.0) and clusterProfiler (version 4.10.0) packages.

### Pseudotime Trajectory Analysis

CytoTRACE R package (version 0.3.3) was used to predict the differentiation state of cells and absolute development potential for the samples^44^. CytoTRACE scores range from 0 to 1, while higher scores indicate higher stemness (less differentiation). Monocle3 was then used to construct cell trajectory and predict pseudotime^45^. Root cell was identified using the function ‘get_earliest_principal_nod()’ provided in Monocole3. Using the root cell (marked with cell “1”) as a starting point, we constructed the cell trajectory and predicted pseudotime.

### Cell culture for Mitophagy studies

U2-OS cells were purchased from ATCC and cultured in RPMI 1640 medium supplemented with 10% (v/v) FBS and 200 units/mL penicillin-streptomycin^46^. TERT2-immortalized HUVEC cells were purchased from Evercyte, Austria (Cat. CHT-006–0008) and were grown in EBM basal medium (Lonza, Cat#CC-3121) supplemented with Components of EGM SingleQuot Kit (Lonza, Cat# CC-4133: BBE, hEGF, hydrocortisone, ascorbic acid), 10% FBS, and 20 μg/ml G418. Primary human foreskin fibroblasts (ATCC SCRC-1041) were maintained in primary fibroblast basal medium (ATCC PCS-201–030) supplemented with recommended growth factors and antibiotics (ATCC PCS-201-040). All cell lines were cultured at 37 °C with 5% CO_2_. Cells carrying stable GFP or RFP expression within mitochondria were developed as mentioned before^47^.

### Average mitochondrial area and mitochondrial number analysis

The average mitochondrial surface area was measured in HFF and HUVEC cells expressing soluble GFP within mitochondria, which were developed using previously described protocols from our laboratory^47^. Software and a modified algorithm for measuring mitochondrial size and number were previously described in detail by us^47^. The Pearson’s correlation coefficient was calculated using the coloc2 plug-in of ImageJ.

### Immunofluorescence Microscopy

A detailed protocol for immunofluorescence microscopy has been previously described^48^. The following antibodies were used for IFA. LC3β antibodies (#sc-376404, Santa Cruz Biotechnology) and LC3β antibody (#18725–1-AP, Proteintech).

### Immunoblotting

Immunoblotting was carried out as described before^8,48^. The following antibodies were used for immunoblotting. LONP1 antibody (#ab224316, Abcam), Mitofilin antibody (#ab245764, Abcam), Miga1 (FAM73A) Antibody (#PA553611, Invitrogen), Drp1 antibody (#sc-271583, Santa Cruz Biotechnology), Parkin antibody (#sc-32282, Santa Cruz Biotechnology), Pex14 antibody (#10594–1-AP, Proteintech), p53 antibody (#sc-126, Santa Cruz Biotechnology), LC3β antibody (#18725–1-AP, Proteintech), Tom20 antibody (#sc-17764, Santa Cruz Biotechnology). Equal protein loading was confirmed by Vinculin antibody (#sc-73614, Santa Cruz Biotechnology). All the primary antibodies were used at a dilution of 1:200~1:1000. HRP-conjugated secondary antibodies, which are Goat Anti-Mouse IgG Antibody, HRP-conjugate (#12–349, Sigma-Aldrich) and Goat Anti-Rabbit IgG Antibody, HRP-conjugate (#12–348, Sigma-Aldrich), were used at a dilution of 1:2,000. The raw, uncut blots are shown in Supplementary Figure 2.

### Cytokine ELISA assays

Secreted IL-12 and IL-10 levels were measured in culture media after 10 days of macrophage differentiation. The supernatants were collected and centrifuged at 10,000 g for 15 minutes at 4 °C to remove cellular debris. Cleaned supernatants were aliquoted and frozen at −80 °C. The following kits were used for cytokine measurements: IL-12 p70 ELISA kit (Invitrogen, Thermo Fisher Scientific, Cat# 88-7126-88, Lot# 376132-005) and IL-10 ELISA Kit (Invitrogen, Thermo Fisher Scientific, Cat# 88-7106-88, Lot# 399831-005).

### Statistics

All statistical calculations were performed using GraphPad Prism 10.0. Error bars displayed on graphs represent the means ± SD of three or more independent replicates of an experiment. Statistical significance was calculated separately for each experiment and is described within individual figure legends. For image analysis, six or more biological replicates per sample condition were used to generate the represented data. The results were considered significant at p ≤ 0.05.

### Reporting summary

Further information on research design is available in the Nature Research Reporting Summary linked to this paper.

### Data and Materials availability:

Further information and requests for resources and reagents should be directed to Bhupesh K Prusty (bhupesh.prusty@rsu.lv) and Christopher Rongo (crongo@waksman.rutgers.edu).

### Preparation of K21 for *C. elegans* Experiments

A 50% stock solution of K21 QASD in ethanol (kindly provided by FiteBac LLc., USA) was used to create a working stock colloidal suspension of 0.2% K21 in 0.8% ethanol. An 0.8% ethanol solution without K21 was used as a vehicle-only control. For treatment, agar plates seeded with OP50 were dosed with 150 mL of either K21 or vehicle, and the plate was rotated to ensure the entire surface that contained OP50 was covered in either substance. Plates were then allowed to dry for approximately 20 minutes, allowing the liquid to be absorbed into the surface of the agar. Embryos harvested following bleach isolation were then deposited on the plates and allowed to hatch. Hatched animals were typically assessed once they reached the L4 stage unless otherwise noted.

### Strains and Transgenic Animals

*C. elegans* strains were derived from the N2 strain, with hermaphrodites analyzed in all experiments. Strains, including *lgg-1(tm3489), drp-1(cq5), atfs-1(cmh15), eat-2(ad1116), wwEx53[P*_*acdh-2*_*::gfp], bvIs5[P*_*cyp-35B1*_*::gfp]*, and *zcIs13[P*_*hsp-6*_*::gfp]*, were obtained from the *C. elegans* Genetics Center. Nematodes were cultured on OP50 *E. coli* seeded on NGM plates unless otherwise stated.

The *C. elegans* version of the mitochondrial Keima (*MitoKeima*) sequence was synthesized by GenScript Biotech Corporation (Piscataway, NJ) using a codon-optimized sequence derived from the mammalian mKeima gene that was modified to reflect the preferred codon usage of C. elegans while preserving the original amino acid sequence. To generate the construct *P*_*vha-6*_*::MitoKeima* for expression of MitoKeima in the intestine of C. elegans, a Gateway LR recombination reaction was performed using the donor vector (*pUC57_Mito-mKeimaWormattL*) and the destination vector (*pPS2_pCFJ150_vha-6_GTWY*), which contains the vha-6 promoter. The destination vector was kindly provided by Barth Grant (Rutgers University, NJ, USA). Transgenic strains were generated by microinjecting the *P*_*vha-6*_*::MitoKeima* plasmid (25 μg/μL) along with the co-injection marker *P*_*ttx-3*_*::rfp* (a gift from Oliver Hobert, Columbia Univ.) into the germline of the wild-type N2 strain. To obtain integrated lines, X-ray irradiation was applied to six plates (each containing ~25 worms on a 6 cm NGM plate), followed by standard protocols for isolating stable integrants. The resulting integrated strain, called *odIs167*, was backcrossed to N2 six times prior to use in experiments.

### Epifluorescence Microscopy and Image Analysis

Transgenic strains were grown at 20°C, allowed to lay eggs overnight, and then removed from the plate. K21, vehicle, or ethidium bromide (for examining *P*_*hsp-6*_*::gfp* expression) were added to these plates, which were then monitored until hatched nematodes reached L4 stage. Fluorescent proteins were visualized in nematodes by mounting on 2% agarose pads with 10 mM levamisole.

For MitoKeima analysis, *odIs167[P*_*vha-6*_*::MitoKeima]* transgenic animals were examined via confocal imaging using a spinning disk confocal microscope equipped with a 100× Zeiss Plan-APOCHROMAT oil immersion objective (1.4 NA; Carl Zeiss Microscopy, White Plains, NY), a CREST OPTICS X-Light^™^ V2TP Confocal Imager (Biovision Technologies, Exton, PA), an 89NORTH LDI laser illumination system (89 North, Williston, VT), and a PRIME 95B sCMOS camera (Teledyne Photometrics, Tucson, AZ). Dual-excitation imaging of MitoKeima was achieved using 470 nm and 555 nm excitation wavelengths, with a single emission collected at 620 nm. Mitochondrial morphology and intensity analysis were performed based on the ImageJ macro protocol described by Valente et al.^49^, including rolling ball background subtraction (50 pixel paraboloid), unsharpen mask filtering (2 pixel, weighted 0.6), CLAHE (block size 127, 256 bins, maximum slope of 3.0), and median filtering and despeckle filtering to reduce pixel noise (2.0 pixels). Regions of interest (ROI) and signal thresholding were used to direct the quantification of mean pixel intensity from each of the dual channels. For each identified mitochondria, the ratio of mean pixel intensity emitted at 620 nm from 555 nm excitation (mitophagic) versus 470 nm excitation (non-mitophagic) was calculated and plotted.

Triple labeling experiments to detect mitochondria or lysosomes in nematodes expressing MitoKeima were performed with commercial dyes. To detect mitochondria, transgenic nematodes were stained by incubating them on NGM plates seeded with OP50 and supplemented with 4 μM MitoTrackerTM Green FM (InvitrogenTM by Thermo Fisher Scientific, Cat.#: M7514) for 24 hours. To detect lysosomes, transgenic nematodes were stained with 2 μM LysoTrackerTM Green DND-26 (InvitrogenTM by Thermo Fisher Scientific, Cat.#: L7526) in M9 buffer, incubating them in the dark for 6 hours. Following staining, nematodes were transferred to standard NGM plates for 1 hour in the dark to allow excess dye to clear before imaging.

Starvation was used as positive control for inducing mitophagy in the intestine. Late L4-stage *odIs167* nematodes expressing MitoKeima were first transferred to an unseeded (OP50-free) NGM plate for 1 hour to allow them to cast off food coating their cuticle, then moved to a different unseeded plate and maintained there for 24 hours prior to imaging.

For quantification of GFP-expressing strains, including *wwEx53[P*_*acdh-2*_*::gfp], bvIs5[P*_*cyp-35B1*_*::gfp]*, and *zcIs13[P*_*hsp-6*_*::gfp]* transgenic strains, nematodes were mounted on agarose pads as described above, and the resulting slides were visualized on a AxioImager M1m (Carl Zeiss, Thornwood, NY). A 5X (NA 0.15) or 10X (NA 0.30) A PlanApo objective was used to detect fluorescence. Images were acquired with an ORCA charge-coupled device camera (Hamamatsu, Bridgewater, NJ) by using iVision software v4.1 (Biovision Technologies, Uwchlan, PA). Exposure times were chosen to capture at least 95% of the dynamic range of fluorescent intensity of all samples. Quantification was performed by obtaining outlines of nematodes using transmitted light images. The mean fluorescence intensity within each outline was calculated after subtracting away background coverslip fluorescence using a rolling ball filter in Fiji/ImageJ 2.1.0/1.53c55. All data with normal distributions were analyzed with GraphPad Prism 9.3.0 in most cases using ANOVA with Dunnett’s post-hoc test correction for multiple comparisons.

### Lifespan Analysis

*C. elegans* strains were grown at 20°C on food for at least two generations before the experiments. For synchronization, 20–30 gravid, well-fed adults were transferred to a new NGM plate with live *E. coli* OP50–1 bacteria to lay eggs for 4–5 h, and then removed. Progeny hermaphrodite animals were maintained at 20°C until the late L4 larval stage, and lifespan assays were counted as days after L4 stage. Animals were transferred away from their progeny to fresh OP50-seeded plates every 1–2 day until the end of their reproductive period. Survival analyses were performed using the Kaplan–Meier method, and the significance of differences between survival curves calculated using the log-rank test. Animals that showed defects due to aberrant vulval development or egg laying (e.g. bursting at the vulva, bagging, etc.) or desiccated on the side of the dish were censored at the time of their demise. Three independent biological replicates were performed; all three gave the same mean lifespan.

### OP50 Titration Experiments

Bacterial lawns of OP50 grown on NGM plates were exposed to K21 or vehicle using the same protocol used for *C. elegans* exposure. After 3 days, bacteria were harvested by adding 1.6 mL of sterile M9 buffer to the plates and gently scraping to collect the remaining bacteria. Harvested bacteria were diluted in LB broth over multiple 10-fold serial dilutions, plated on LB plates, and counted for colonies following an overnight incubation at 37°C. Each condition was performed in triplicate.

### Pharyngeal Pumping Analysis

Nematodes were allowed to grow on OP50 lacking drug until L4, when 5–10 were transferred to test plates (either K21 or vehicle) and allowed to acclimate at 20°C for 30 minutes. These plates were then placed under a dissection microscope and recorded using StreamPix SingleCam 10.1.0.0 (x64) under normal monochrome brightfield conditions. Each worm was recorded for approximately 20 seconds, and each video was then observed at 0.25x speed, with each pharyngeal pump recorded on a counting app. This number was then used to calculate the rate per minute for each worm, and then the averages were taken of each condition.

### OP50 Plate Clearance Analysis

Feeding behavior was assessed by measuring the optical density at 600 nm (OD_600_) of *E. coli* OP50 cultures using a Tecan Infinite^®^ M200 Pro plate reader with the Tecan i-control software. Greiner 96-well flat-bottom transparent plates were used for analysis. Bacterial lawns of OP50 grown on NGM plates were harvested by adding 1.6 mL of sterile M9 buffer to the plates and gently scraping to collect the remaining bacteria. Under these conditions, OD_600_ values for control and K21-treated plates without nematodes were approximately 1, while the blank M9 buffer had an OD_600_ value of approximately 0.04. For OD_600_ measurements, 300 μL of the sample was loaded per well, followed by 12 seconds of linear shaking at 1.5 mm amplitude prior to readings. Each well was measured using 20 flashes with triplicate reads (3 × 3 points per well), and the average value was recorded for each sample. A standard curve was generated using serial dilutions of harvested bacteria (concentrations: 1, 0.75, 0.5, 0.25, 0.125, and 0), prepared 24 hours prior to the experiment, and OD_600_ values were recorded for each dilution. Following generation of the standard curve, 200 hand-picked young adult stage *C. elegans* N2 and *eat-2(ad1116)* animals, grown from eggs under identical conditions, were added to both control and K21-treated plates. Food consumption rates were assessed by measuring the OD_600_ of the remaining bacteria 24 hours after introducing the worms. Each condition was performed in triplicate.

### *C. elegans* Growth and Development Assays

All genotypes were synchronized by alkaline bleaching and arrested at L1 stage overnight in M9 buffer. Synchronized genotypes were assayed 43–46 hours after reaching the L4 stage, when embryos present within adult animals were counted using a dissection microscope. From 40–60 animals were analyzed and pooled from three biological replicates. Averages represent unlaid eggs per animal.

### RNA-seq

Developmentally synchronized animals were obtained by hypochlorite treatment of gravid adults and embryos hatched overnight for 15–17 hours in M9. Starvation-arrested L1s were plated on NGM plates and grown at 20 °C until L4 stage. Total RNA was isolated from animals using Trizol (Invitrogen) combined with Bead Beater lysis in five biological replicates for each genotype. RIN values for the RNA ranged from 9.4–9.9. An mRNA library (paired-end, 150-bp reads) was prepared for each sample/replicate using Illumina Truseq with PolyA selection (Azenta/GeneWiz). Libraries were sequenced across two lanes on an Illumina HiSeq2500 (Azenta/GeneWiz), resulting in a range of 27–37M reads per sample, with peak mean phred scores of 38.76. Reads were mapped to the *C. elegans* genome (WS245) and gene counts generated with STAR 2.5.1a. Normalization and statistical analysis on gene counts were performed with EdgeR using generalized linear model functionality and tagwise dispersion estimates. Multidimensional scaling analysis showed tight clustering within five biological replicates, with a clear separation between treatments. Likelihood ratio tests were conducted in a pairwise fashion between genotypes with a Benjamini and Hochberg correction. Genes were considered to be differentially regulated if they were differentially expressed with an FDR<0.01 in the same direction (up or down). RNA-seq data sets are available at NIH/NCBI GEO through accession number GSE296571.

### Gene Ontology and Gene Set Enrichment Analysis (GSEA)

For gene ontology enrichment, three separate sets of differentially expressed genes from the RNA-seq analysis were analyzed: “UP” genes, which were genes showing elevated expression following K21; “DOWN” genes, which were genes showing depressed expression following K21; and “ALL” genes, which were a combination of the UP and DOWN genes. Gene ontology enrichment was performed using two separate approaches. WormCat 2.0 (http://www.wormcat.com) and gProfiler annotational clustering (https://biit.cs.ut.ee/gprofiler/gost)^50,51^.

For testing enrichment of user-defined gene lists, Gene Set Enrichment Analysis (GSEA) was performed on DEGs from the RNA-seq. GSEA version 4.3.3 [build:16] was used with the preranked RNA-seq data to determine whether there was enrichment for specific stress response pathways as described by Soo et al^22^. These experiments were run using the preranked version of the software, and the permutations were set at the default 1000.

### Orthology Analysis

Differentially expressed genes (FDR 0.05) induced by K21 treatment in the macrophage and monocyte populations as identified in the scRNA-seq were used as queries in Ortholist2 (http://ortholist.shaye-lab.org) to identify putative orthologs based on matches in at least two Ortholist2 databases. Identified *C. elegans* orthologs were then crossreferenced with a strict threshold (FDR 0.001) of DEGs from the *C. elegans* RNA-seq induced by K21 treatment. Log2 fold changes from the PBMC scRNA-seq and *C. elegans* RNA-seq for each *C. elegans*/Human ortholog combination were then visualized in heat map and organized based on presence or absence in overlapping datasets.

### Statistical Analysis

Simple calculations were done in MS Excel v16.64. Data for phenotypic analysis (e.g., survival assays, lifespan assays, fertility assays, individual animal fluorescence measurements) were analyzed using GraphPad Prism 9.3.0. Larger datasets (e.g., RNA-seq) were analyzed with scripts written in R or as otherwise indicated above. Where possible, researchers were blind to genotype or experimental treatment. Statistical tests were as indicated in figures; two-sided tests were used unless otherwise indicated. Data was tested for normality using Kolmogorov-Smirnov and adjusted for multiple comparisons as indicated in the figure legends.

## Supplementary Material

Supplementary Files

This is a list of supplementary files associated with this preprint. Click to download.

- SupplementaryInformationGuide.docx
- SupplementaryFigure1.pdf
- SupplementaryFigure2.pdf
- ExtendedDataFigure1v2.pdf
- ExtendedDataFigure2.pdf
- ExtendedDataFigure3.pdf
- ExtendedDataFigure4.pdf
- ExtendedDataFigure5.pdf
- ExtendedDataFigure6.pdf
- ExtendedDataFigure7.pdf
- ExtendedDataFigure8.pdf
- ExtendedDataFigure9.pdf
- ExtendedDataFigure10.pdf
- ExtendedDataFigure11.pdf
- ExtendedDataFigure12.pdf
- ExtendedDataFigure13.pdf
- ExtendedDataFigure14.pdf
- ExtendedDataFigure15.pdf
- ExtendedDataFigure16.pdf
- ExtendedDataFigure17.pdf
- ExtendedDataFigure18.pdf
- ExtendedDataFigure19.pdf
- ExtendedDataFigure20.pdf
- ExtendedDataFigure21.pdf

## Data Availability

Filles for the human monocyte-derived macrophage scRNA-seq data sets are available at the NHI/NCBI GEO under accession numbers GSE301383, and the same for PBMC scSLAM-seq data GSE301385. Files for the *C. elegance* RNA-seq data sets are available at the NIH/NCBI GEO under accession number GSE296571. Files can be directly accessed at the web link https://www.ncbi.nlm.nih.gov/geo/query/acc.cgi?acc=GSE296571. The files GSM8972749, GSM8972750, GSM8972751, GSM8972752, and GSM8972753 contain data for five independent biological replicates for N2 wild-type nematodes treated with K21. The files GSM8972744, GSM8972745, GSM8972746, GSM8972747, and GSM8972748 contain data for five independent biological replicates for N2 wild-type nematodes treated with vehicle alone. Other source data are provided online as a single Source Data with this paper.
